# A Novel Nonsense Variant in *GRM1* Causes Autosomal Recessive Spinocerebellar Ataxia 13 in a Consanguineous Pakistani Family

**DOI:** 10.3390/genes13091667

**Published:** 2022-09-17

**Authors:** Hammad Yousaf, Ambrin Fatima, Zafar Ali, Shahid M. Baig, Mathias Toft, Zafar Iqbal

**Affiliations:** 1 Human Molecular Genetics Laboratory, National Institute for Biotechnology and Genetic Engineering College (NIBGE-C), Pakistan Institute of Engineering and Applied Sciences (PIEAS), Islamabad 44000, Pakistan; 2 Department of Biological and Biomedical Sciences, The Aga Khan University, Karachi 74800, Pakistan; 3 Centre for Biotechnology and Microbiology, University of Swat, Swat 01923, Pakistan; 4 Department of Neurology, Oslo University Hospital, P.O. Box 4950 Nydalen, N-0424 Oslo, Norway; 5 Institute of Clinical Medicine, University of Oslo, P.O Box 1171, N-0318 Oslo, Norway

**Keywords:** SCAR13, *GRM1*, nonsense variant, familial ataxia, Pakistan, exome sequencing, metabotropic glutamate receptor 1

## Abstract

**Background and objectives:** Autosomal recessive spinocerebellar ataxia-13 (SCAR13) is an ultra-rare disorder characterized by slowly progressive cerebellar ataxia, cognitive deficiencies, and skeletal and oculomotor abnormalities. The objective of this case report is to expand the clinical and molecular spectrum of SCAR13. **Methods:** We investigated a consanguineous Pakistani family with four patients partially presenting with clinical features of SCAR13 using whole exome sequencing. Segregation analysis was performed by Sanger sequencing in all the available individuals of the family. **Results:** Patients presented with quadrupedal gait, delayed developmental milestones, non-progressive peripheral neuropathy, and cognitive impairment. Whole exome sequencing identified a novel pathogenic nonsense homozygous variant, Gly240*, in the gene *GRM1* as a cause of SCAR13 that segregates with the recessive disease. **Discussion:** We report a novel homozygous nonsense variant in the *GRM1* gene in four Pakistani patients presenting with clinical features that partially overlap with the already reported phenotype of SCAR13. In addition, the family presented quadrupedal gait and non-progressive symptoms, manifestations which have not been recognized previously. So far, only four variants in *GRM1* have been reported, in families of Roma, Iranian, and Tunisian origins. The current study adds to the mutation spectrum of *GRM1* and provides a rare presentation of SCAR13, the first from the Pakistani population.

## 1. Introduction

Autosomal recessive spinocerebellar ataxias (SCARs) encompass a large group of clinico-genetically heterogeneous entities with overlapping clinical presentations causing diagnostic complications [1]. The key clinical features of SCARs include, but are not limited to, the deterioration of cerebellum, brain stem, spinal cord, and nerves, manifesting as developmental delay, gait abnormalities, and intellectual disability. Therefore, differential diagnosis is challenging to establish. These disorders are also associated with other variable non-neurological multisystem deficits characteristic of each subtypes [2,3].

SCAR13 (MIM#614831) is an ultra-rare subtype caused by the disruptive mutations in the glutamate metabotropic receptor 1 *(GRM1)* gene. Only four biallelic variants in 14 individuals have been reported to cause SCAR13 in the literature [3,4,5]. In 2012, Guergueltcheva et al. reported for the first time two biallelic variants segregating with clinical features in ten patients from five Roma families [3]. Three years later, Davarniya et al. reported another biallelic variant in three Iranian patients of a family suffering from SCAR13 [5]. In 2019, Cabet et al. reported a biallelic truncating variant in a patient of Tunisian origin [4]. In all these reports, patients had slowly progressive cerebellar ataxia, variable cognition impairment, motor delay, and skeletal and oculomotor abnormalities.

*GRM1* encodes G-protein-coupled metabotropic glutamate receptor 1 (mGluR1), which is highly expressed in cerebellar Purkinje cells [6,7]. It expresses primarily at postsynaptic densities and performs through activation of phospholipase C and formation of inositol 1,4,5-triphosphate/diacylglycerol [8,9,10]. *GRM1* plays a key role in cerebellar development, cognition, and neuroprotection through activation of second messenger systems, thus maintaining synaptic plasticity [11].

The recent technological advances in genomics through next-generation sequencing (NGS) and the availability of highly efficient curation algorithms have improved our diagnostic abilities for these disorders. In the last decade, >100 novel SCARs genes have been discovered and mutations in the majority of these genes are ultra-rare [12]. With the addition of novel clinical/genetic information, the field is progressing towards a refined classification of SCARs, leading to better understanding and diagnosis. In the same context, we herewith report a novel nonsense recessive variant, Gly240*, in *GRM1* in a multiplex Pakistani family with four affected individuals with rare presentation of SCAR13, being the first one from Pakistan. Thus, we provide further evidence on *GRM1*’s implication in early cerebellar development and maintenance.

## 2. Methods and Results

### 2.1. Case Report

The current study was conducted following the rules of the Declaration of Helsinki and was approved by the ethics committee of the National Institute for Biotechnology and Genetic Engineering College (NIBGE-C), Faisalabad, Pakistan. We studied a consanguineous family originating from Mardan, Pakistan. Four male patients (IV:1–IV:4, Figure 1A,B) presented with a constellation of non-progressive symptoms comprising gait ataxia scaled to quadrupedal gait associated with nodding (videos available on request from corresponding author), severe motor delay, intellectual disability, and ocular and skeletal abnormalities.

Patients were aged from 17 to 29 years at the time of examination. Patients were born at term by normal vaginal delivery. The gestational and birth histories of the patients were uneventful. Clinical information of the patients is summarized in Table 1. Symptoms were noticed as early as the seventh day after birth by a lack of neonatal reflexes. Parents mentioned that the patients remained unresponsive to their surroundings, loud noises, and abrupt movements in their infancy (Moro reflex). Patients also manifested feeding problems associated with rooting or sucking reflex abnormalities.

A marked delay in achieving developmental milestones was observed. Patients were unable to hold their head until the age of 3 years. Sitting was achieved around 4.5 years, and a year later patients started to crawl. Standing and walking were never attained, and patients are still dependent for self-care and feeding. Gait ataxia and profound intellectual disability persisted as stationery manifestations with no signs of progression. Patients are unable to speak but can make meaningless sounds and are able to socialize through eye contact and nonspecific hand movements. Unable to communicate verbally, patients are mildly responsive to nonverbal communication cues. We evaluated severity of ataxia by using “the Scale for the Assessment and Rating of Ataxia (SARA)” [13] and scores are mentioned in Table 1. We noticed severe gait, stance, and speech disturbances in all individuals (18/18 score). Moderate to severe dysmetria, tremors, and dysdiadochokinesia (SARA items 5–8; maximum score of 16) were present with a mean score of 11.5. Ophthalmological examination revealed consistent eye ptosis and strabismus in all patients. Intriguingly, individual IV:2 exhibited spinal curvature dysmorphism in the form of scoliosis. Patients are short-tempered and are triggered even by trivial reasons, leading to aggression. As already described, we also noticed mild corticospinal signs in the form of the Babinski sign and hyperreflexia. We also observed myopia in the patients. Moreover, we did not observe any neurological phenotype in parents. Due to the unfavorable circumstances, we were unable to get further clinical information through brain MRI scans.

### 2.2. Molecular Diagnosis

After obtaining ethical approval and informed consent, genomic DNA was extracted from the peripheral blood of the patients and the available parent (III:1, mother) using a protocol already described [14]. Whole exome sequencing (WES) of the proband IV:1 was carried out at Novogene Co., Ltd (Cambridge, UK). In brief, Agilent SureSelect Human All Exome V6 (Agilent Technologies, Santa Clara, CA, USA) was used to capture the whole exome and subsequent paired-end (PE150) sequencing was performed on an Illumina platform, NovaSeq 6000 (Illumina, Santa Clara, CA, USA). The sequencing reads were mapped to the reference genome (hg19) with Burrows-Wheeler Aligner v0.7.17 (BWA) [15]. SAMtools [16] v1.8, and Picard v2.18.9 (http://sourceforge.net/projects/picard/) were utilized to sort BAM files and to mark duplicate reads, respectively. Genotyping was performed with Genome Analysis Toolkit v4.0 (GATK) [17]. Functional annotation of the variants was carried out with Annotate Variation (ANNOVAR) [18]. Variant filtering was carried out with FILTUS [19].

We used several parameters for the filtration process of the variants. We considered missense and nonsense variants, InDels, and variants at the canonical splice sites and excluded variants with minor allele frequency (MAF) greater than 0.01 in publicly available resources: the Exome Sequencing Project (ESP, https://evs.gs.washington.edu/EVS/), 1000 Genomes Project (https://www.internationalgenome.org/), and Genome Aggregation Database (gnomAd) (https://gnomad.broadinstitute.org/). We also excluded the variants with Combined Annotation Dependent Depletion (CADD) [19,20] scores lower than 10. Further, we considered pathogenic and likely-pathogenic variants and used the variant classification system set out by the American College of Medical Genetics and Genomics (ACMG) [21]. Candidate Variants were visually inspected with the Integrative Genomics Viewer (IGV, https://software.broadinstitute.org/software/igv/) to remove any artefacts and false positives.

**Figure 1:**
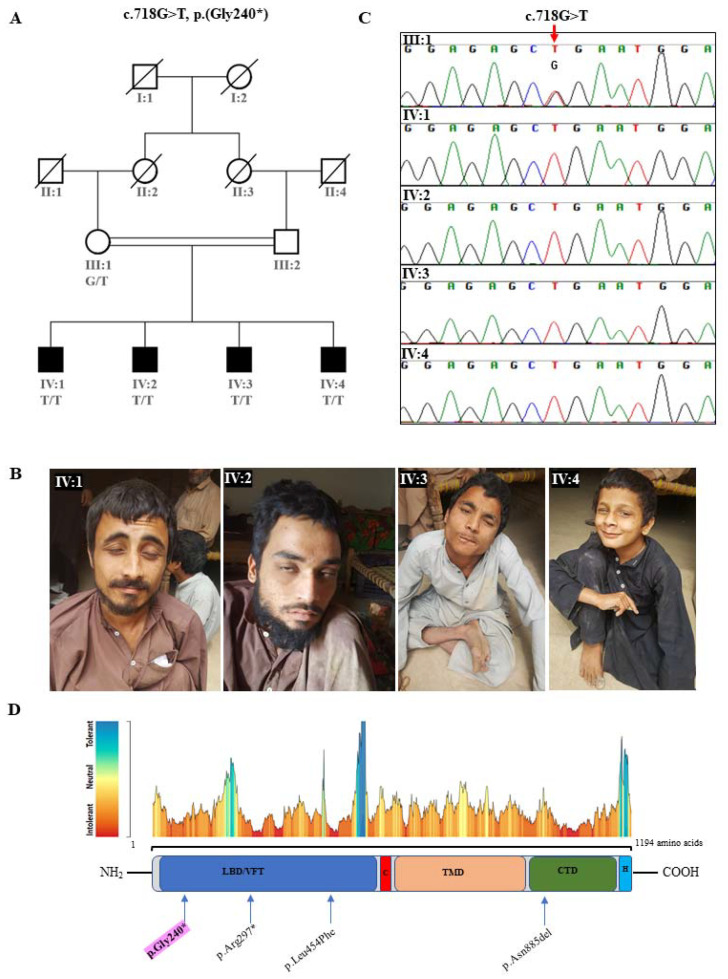
(**A**) Four-generation pedigree of the family showing four affected males (filled squares) born to first cousins. All affected individuals were homozygous (T/T) while the mother was carrier for the variant (G/T). (**B**) Representative images of four affected individuals (IV:1–4). (**C**) Chromatograms showing the *GRM1:* c.718G>T: p. (Gly240*) variant specified by red arrow on the top. (**D**) The domain structure of the 1194 amino acid mGluR1α isoform obtained from [5,9,24,25,26] and the NCBI “Conserved domain search” tool. LBD/VFT: ligand-binding domain/Venus fly trap; C: cysteine-rich domain; TMD: heptaspanning transmembrane domain; CTD: C-terminal domain; H: Homer 1 binding motif. Pink highlighted variant is the novel variant identified in the present study. Variants in black are known to cause SCAR13 [3,4,5]. The figure shows all protein-affecting variants in *GRM1*, except a splicing variant c.2660+2T>G [3]. Top region of the figure represents intolerance to variation landscape at every amino acid position of *GRM1* [25].

Our WES analysis pipeline revealed a novel pathogenic homozygous nonsense variant (c.718G>T: p. (Gly240*)) in the gene *GRM1* (GenBank: NM_001278064.2). The list of filtered variants in the family is available in Table S1. The variant *GRM1*:Gly240* is absent in the gnomAd database (accessed on 2 June 2022) and has a CADD score of 40 (CADD model GRCh38-v1.6) [22]. The identified pathogenic variant triggered PVS1 (very strong), PM2 (moderate), and PP3 (supporting) rules of ACMG criteria for variant interpretation [21]. Sanger sequencing revealed the segregation of the novel pathogenic variant with the phenotype and confirmed an autosomal recessive pattern of inheritance, as the mother is a heterozygous mutation carrier while patients are in the homozygous mutant state (Figure 1C).

## 3. Discussion

The current study was aimed to find the plausible genetic variant in an inbred Pakistani family with four patients suffering from motor, learning, and coordination deficiencies. The present study is the first instance of SCAR13 being reported in patients of Pakistani ethnicity. The partially novel clinical features of the patients in this study are listed in Table 1 that partly overlap with the already reported phenotype of SCAR13 (summarized by Cabet et al., 2019 [4]). Quadrupedal gait associated with nodding is reported for the first time in patients with SCAR13. Non-progression of the disease was observed, which is a disparity with the reported phenotype. Another interesting fact is that the age of onset in our cases was the seventh day after birth, which could be the earliest notice to caretakers and healthcare providers for a vigilant follow-up for timely management. Absence of neonatal reflexes, such as Moro and sucking/ rooting, point towards defective neurological development, potentially due to a severe disturbance in the mGluR1 signaling pathway. Physiologically, these neonatal reflexes should be present at birth and vanish as the voluntary motor skills develop over time. We did not observe any history of seizures, which was reported in 5/14 patients previously [3,4,5]. Furthermore, we did not observe any other dysmorphism in our patients.

We report a novel variant Gly240* in the *GRM1* gene introducing a premature protein truncation, assuming a loss of function mechanism. So far, variants reported in *GRM1*-SCAR13 are nonsense, missense, splicing, and indels [3,4,5], spanning the ligand-binding domain, transmembrane domain, and c-terminal domain of the protein. Due to the inaccessibility of the patients’ relevant material, it was not possible to determine the exact consequence of the identified nonsense variant on the integrity of the resultant protein. We can, however, predict three possible scenarios here: (1) no mRNA is produced due to the nonsense-mediated RNA decay and, consequently, complete loss of the protein; (2) a truncated and unstable protein is produced, leading to complete loss of the protein; or (3) a truncated protein is produced, lacking most of the necessary functionalities. Considering the latter situation, the variant Gly240* lies in the ligand-binding domain of mGluR1, leading to the truncated polypeptide chain devoid of the functional signal transmission domains (i.e., hepta-spanning transmembrane and downstream domains). Thus, the physiological roles in long-term potentiation in the hippocampus and long-term depression in the cerebellum will possibly be lost [5,8,23].

mGlur1 executes its functions as paired transmembrane signal transducers that activate primarily through binding of the excitatory neurotransmitter glutamate. This 1194 amino-acid-long peptide spans a “ligand binding domain”, also called the “Venus fly trap” domain, followed by a “cysteine rich domain”, a conserved “hepta-spanning transmembrane domain”, and a “c-terminal G-protein coupled intracellular domain” trailed by a “homer binding motif” [5,9,24,25,26]. Binding of the long Homer proteins (Homer 1b, 1c, 2b, and 3) to this domain initiates a series of conformational changes, resulting in plasma membrane clustering/dimerization for efficient signaling [27]. Upon binding of the glutamate, the signaling cascade terminates with the release of intracellular calcium ions [3,4,24,26]. The protein domain structure of mGlur1α (major isoform out of five isoforms) is represented in Figure 1D, along with the position of SCAR13 implicated variants in *GRM1* [3,4,5]. The pertinent role of mGluR1 signaling is reflected by the intolerance to variation landscape plotted by MetaDome [25], showing most of the amino acids in the red to orange zone (i.e., highly intolerant to slightly intolerant), as shown in top region of Figure 1D.

The role of mGlur1 signaling in neurotransmission has also been demonstrated in knockout mice. The Mouse Genome Database of the Jackson Laboratory [28] reports that homozygous knockout mice for null variants showed uncoordinated motor functions, hippocampal long-term potentiation and cerebellar long-term depression anomalies resulting in a complex ataxic phenotype, along with developmental/functional cerebellar deficits associated with learning disabilities and skeletal dysmorphism exhibiting kyphoscoliosis [6,29,30,31,32]. Interestingly, one of our patients (IV:2) showed scoliosis, which has not been recognized in SCAR13 patients previously, emphasizing that more efforts are required to dissect the clinical and molecular basis of SCAR13.

To date, there is no cure available for SCAR13. In a mice model of SCAR13, it was observed that homozygous *Grm1^crv4/crv4^* mice lacking mGluR1 receptors had overexpression and activation of mGluR5, which was proposed to cause the ataxic phenotype [31]. To prove this, Bossi et al., 2018 [33] generated double mutants (*Grm1^crv4/crv4^Grm5^ko/ko^*) and showed the improvement in ataxic phenotype. Although not studied in detail so far, it could be a plausible therapeutic target in patients with defective mGluR1-mediated ataxia.

Here, we further reinforce the role of disrupted mGluR1 signaling affecting cerebellar function that could lead to neonatal onset SCAR13. Thus far, only 14 patients and 4 variants have been reported, from families of Roma, Iranian, and Tunisian origins. This study raised the total number of SCAR13 implicated variants in *GRM1* to 5, spanning the 18 patients reported so far. The importance of this study includes the report of a partially novel phenotype of SCAR13; the identification of a novel pathogenic *GRM1* disease variant; and, finally, the novel ethnicity of the family. We also recommend a more uniform criterion for collecting clinical information to achieve a consensus on the phenotypic spectrum of SCAR13. This would aid clinicians in timely diagnosis and management through physiotherapy and rehabilitation efforts and could help identify the families for cascade testing to reduce the disease burden through prenatal genetic testing.

## Figures and Tables

**Table 1:** Summary of clinical features of the patients in this study.

	Patients			
	IV:1	IV:2	IV:3	IV:4
Gender	Male			
Age (years)	29	22	20	17
**Developmental Milestone**				
Sitting (years)	4.5	4.5	4.5	4.5
Crawling (years)	5.5	5.5	5.5	5.5
Standing	-	-	-	-
Walking	-	-	-	-
Single work	-	-	-	-
Self-care	-	-	-	-
**Cerebellar Ataxia (SARA scores) ^a^**				
Gait (0–8)	8	8	8	8
Stance (0–6)	6	6	6	6
Sitting (0–4)	0	0	0	0
Speech distrubance (0–6)	6	6	6	6
Finger chase (L + R)/2 (0–4)	2	3	4	2
Nose-finger test (L + R)/2 (0–4)	2	3	4	2
Fast alternating hand movements (L + R)/2 (0–4)	2	3	4	2
Heel-shin slide (L + R)/2 (0–4)	3	3	4	3
Total SARA score (0–40)	29/40	32/40	36/40	29/40
**Neurological Signs**				
Ataxia	Quadrupedal	Quadrupedal	Quadrupedal	Quadrupedal
Dysarthria	+	+	+	+
Babinski sign	+	+	+	+
Hyperreflexia	+	+	+	+
Dysmetria	+	+	+	+
Intellectual disability	Severe	Severe	Severe	Severe
Aggressive behavior	+	+	+	+
Clinical progression	-	-	-	-
Seizures	-	-	-	-
Hypotonia	-	-	-	-
Brain abnormalities (MRI)	ND	ND	ND	ND
**Ophthalmological Abnormalities**				
Eye ptosis	+	+	+	+
Strabismus	+	+	+	+
**Skeletal Abnormalities**				
Spine curvature deformity	-	Scoliosis	-	-
Facial dysmorphism	-	-	-	-
Pes planus	+	+	+	+

^a^ Scale for the Assessment and Rating of Ataxia. Eight items were scored. Numbers in the brackets after each item show the severity, with 0 indicating no impairment and higher scores indicating increasing severity. Abbreviations: left (L) and right (R). Legend: presence (+) or absence (-) of features; ND: not determined.

## Data Availability

The data from this study can be made available upon request from the corresponding author.

## Supplementary Materials

The following supporting information can be downloaded at: https://www.mdpi.com/article/10.3390/genes13091667/s1, Table S1: List of filtered variants in this family.
